# Identification of Alzheimer’s Disease by Imaging: A Comprehensive Review

**DOI:** 10.3390/ijerph20021273

**Published:** 2023-01-10

**Authors:** Prasath T., Sumathi V.

**Affiliations:** 1 School of Electrical Engineering, VIT Chennai, Chennai 600127, Tamil Nadu, India; 2 Centre for Automation, School of Electrical Engineering, VIT Chennai, Chennai 600127, Tamil Nadu, India

**Keywords:** medical imaging, image segmentation, image registration, image reconstruction, Alzheimer’s disease, non-invasive method

## Abstract

In developing countries, there is more concern for Alzheimer’s disease (AD) by public health professionals due to its catastrophic effects on the elderly. Early detection of this disease helps in starting the therapy soon and slows down the progression of the disease. Imaging techniques are considered to be the best solutions for its detection. Brain imaging was initially used to diagnose AD. Different techniques for identifying protein accumulation in the nervous system, a sign of Alzheimer’s disease, are identified by MRI imaging. Although they were initially attributed to cortical dysfunction, visual system impairments in Alzheimer’s patients were also found in the early 1970s. Several non-invasive approaches reported for screening, prevention, and therapy were unsuccessful. It is vitally necessary to develop new diagnostic methods in order to accurately identify patients who are in the early stages of this disease. It would be wonderful to have a quick, non-invasive, affordable, and easily scalable Alzheimer’s disease screening. Researchers may be able to identify biomarkers for Alzheimer’s disease and understand more about its aetiology with imaging and data processing. This study clarifies the need for medical image processing and analysis strategies which aid in the non-invasive diagnosis of AD.

## 1. Introduction

Alzheimer’s disease is a brain disorder that slowly destroys memory and thinking skills and, eventually, the ability to carry out simple tasks. Image processing techniques are frequently used to assist physicians in making observation-based decisions when diagnosing illnesses such as Alzheimer’s disease. AD affects one in twenty-six people globally according to studies. Accumulation of amyloid plaques and neurofibrillary tangles, which characterize Alzheimer’s disease from a neuropathological perspective, result in the gradual deterioration of memory, learning, and executive function. Alzheimer’s disease is an incurable, degenerative, and ultimately fatal condition that affects people and shows signs only in the later stage.

“Disease-modifying” experimental medicines have not been developed for individuals with Alzheimer’s disease. The most plausible reason for these failures is that the administration of the drug at a point in the disease’s neuropathology is considered to be late [1]. Due to this, the development of biomarkers that detect Alzheimer’s disease in its preclinical or primitive clinical phases is critical for the success of these medicines. Therapy testing for Alzheimer’s disease is still in its primitive phase; therefore, a better outcome is yet to be confirmed, and earlier treatment may improve outcomes [2]. Additionally, primitive detection enables the development of effective treatment due to its ease of monitoring. Patients and their families benefit from a primitive diagnosis because it gives them the opportunity to learn about the disease, weigh the financial and personal implications, and prepare for the patient’s long-term needs and treatment options. 

Currently, this condition can be diagnosed through medical examinations, cognitive tests, magnetic resonance imaging (MRI), cerebrospinal fluid analysis, and blood tests. This process is inefficient in terms of both time and money. Due to the increase in demand for Alzheimer’s disease treatment, there are not enough specialized hospitals in existence to meet the demand. Developing clinically applicable screening and diagnostic methods is a key driving factor because medical professionals are unable to detect primitive Alzheimer’s disease or moderate cognitive impairment (MCI) [3]. Physical activity in patients has been studied as a treatment for pre-clinical Alzheimer’s disease, and late-stage Alzheimer’s disease, and as a preventative strategy with varying drug success. Results reveal an increase in hippocampus volume, better neurogenesis, and improved blood flow. It is inferred from growing research studies that exercise lowers the risk of Alzheimer’s disease.

### 1.1. Image Processing and Its Applications in Disease Detection

Imaging of the human body in three dimensions produced from a CT (computed tomography) or MRI scanner is routinely used to diagnose disease, guide medical treatments such as surgery, or simply for research purposes in the field of medical image processing. Many methods, including as segmentation and texture analysis, are used in medicine to help detect cancer and other diseases. Modern imaging modalities, such as PET-CT (positron emission tomography-CT) and PET-MRI, make extensive use of image registration and fusion algorithms. To keep tabs on the course of a continuing sickness, medical imaging is a must. Physicians track the progress of their patient’s treatment plans and make appropriate adjustments based on the results of imaging tests such as MRIs and CT scans. Patients benefit from complete treatment due to the sufficient data produced by medical imaging and image processing techniques, which aid in detecting malignancies in MRI images. Visual field (VF), optical disc (OD), optical coherence tomography (OCT), and optical coherence tomography angiography (OCTA) are utilised in the diagnosis of retinal diseases such as macular degeneration, glaucoma, and AD.

### 1.2. Evidence of Alzheimer’s Disease Influencing Central Nervous System

**Figure 1:**
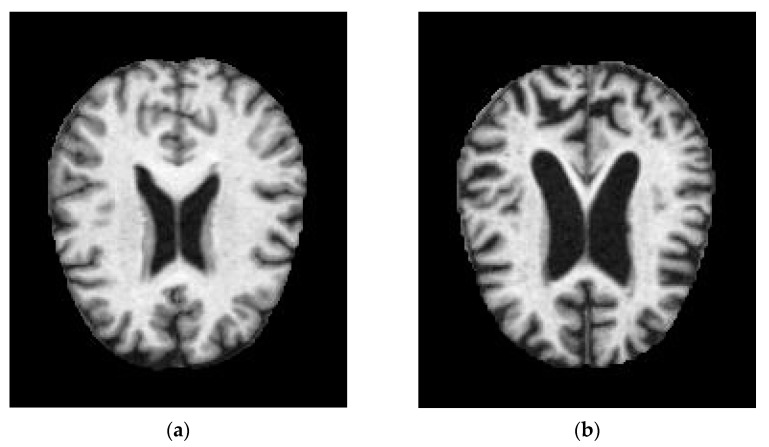
MRI images of (**a**) normal demented and (**b**) moderated demented.

Alzheimer’s disease begins its slow progression in the central nervous system. AD is characterized by the breaking of neuronal connection links, the damage of tissue cells, and the shrinking of brain regions. Amyloid plaques and tangles accumulation in the brain are also considered to be the features of AD. Various methods were used to detect the early stages of Alzheimer’s disease. Imaging techniques are frequently used in Alzheimer’s disease diagnosis. MRI and positron emission tomography (PET) studies of cerebral metabolism through a variety of imaging techniques have shown characteristic changes in AD patients. MRI image of a normal demented and moderate demented is shown in Figure 1. Severity conditions can also be detected by comparing the MRI with a healthy brain image. As the number of patients increases, early detection might help in the diagnosis, which has been difficult for physicians. To address this, based on the available MRI dataset of AD patients, machine learning and deep learning techniques of artificial intelligence were utilized. Various approaches have been deployed so far for the detection of AD using artificial intelligence; the estimated time of detection and accuracy are considered to be important. AD shows its early sign in the visual system which can be identified by the continuous progression of retinal fibres. Developing a non-invasive method for diagnosing AD can ease patients’ concerns which can be accomplished through retinal imaging.

### 1.3. Evidence of Alzheimer’s Disease Influencing the Visual System at the Molecular Stage

The retinal nerve fibre layer (RNFL) and optic nerve serve as the brain’s principal window following development since their axons are directly connected to multiple brain regions. In certain studies, the retina and optic nerve contain molecular indicators for Alzheimer’s disease [4,5]. Deposits of amyloid-β (Aβ) and hyperphosphorylated tau, which form SPs (senile plaques) and NFTs (neurofibrillary tangles) in brain regions such as the visual cortex are thought to cause neurotoxic damage and cell death. SPs and NFTs may cause RGC mortality, RNFL thinning, and optic nerve structural anomalies in Alzheimer’s disease patients’ retina. Neuroretina and retinal vasculature are affected by altered amyloid precursor protein (APP) processing and fibrillar Aβ deposition in transgenic Alzheimer’s disease animal models [5,6]. For those suffering from Alzheimer’s disease, it might be difficult to distinguish between primitive-phase vision problems and symptoms of the disease due to the presence of SP and NFT deposits in their visual cortex and neuroretina. Curcumin, a fluorochrome for plaque labelling that has not previously been studied, generates plaques in the retina, but this is before plaques reach the brain, as demonstrated by Koronyo-Hamaoui et al. [4]. In this way, the accumulation of Aβ in ganglion cells may serve as a primitive warning symptom of Alzheimer’s disease. 

Modern literature [7,8] suggests that soluble oligomers of Aβ accumulate in synapses before fibrillar Aβ deposition and neurodegeneration. The retina has also been found to contain Aβ oligomers [9]. Neuronal apoptosis is thought to be produced by Aβ oligomers in Alzheimer’s disease by RGC (retinal ganglion cell) loss such as Aβ oligomers in animal models. Drusen, the extracellular deposits that age-related macular degeneration (ARMD) is known for, contain soluble Aβ oligomers, but their role in atrophy in ARMD has not been established yet. P301S tau mutation in a transgenic mouse model has been associated with axonal transport disruption and ganglion cell degeneration [10]. RNFL hyperphosphorylation was seen in this model. Alzheimer’s disease eye impairment may be caused by tau and APP (amyloid precursor protein) according to a number of studies. It may be easier in the future for the issue of discrimination between Alzheimer’s disease and visual disorders. 

### 1.4. Neurochemistry Deficiency in the AD Retina

Pathogenesis of Alzheimer’s disease includes acetylcholine deficiency [11]. Presynaptic degeneration is seen in cholinergic pyramidal neurons. Though many excitatory neurotransmitters (such as glutamate and noradrenaline) have been related to Alzheimer’s disease, cholinergic transmission is the one that is most impacted. Acetylcholine activates muscarinic receptors on ganglion cells in the retina, resulting in cholinergic signalling [12,13]. While Alzheimer’s disease patients’ retina shows no indication of impaired cholinergic transmission, neuronal feedback mechanisms are nonetheless responsible for retinal acetylcholine activity. Strenn et al. [14] examined the electroretinogram patterns, scotopic brightness, and photopic brightness in Alzheimer’s patients and age-matched healthy controls and found no evidence of neurochemical retinal deficiency in the illness.

## 2. Non-Invasive Imaging of the Retina

**Figure 2:**
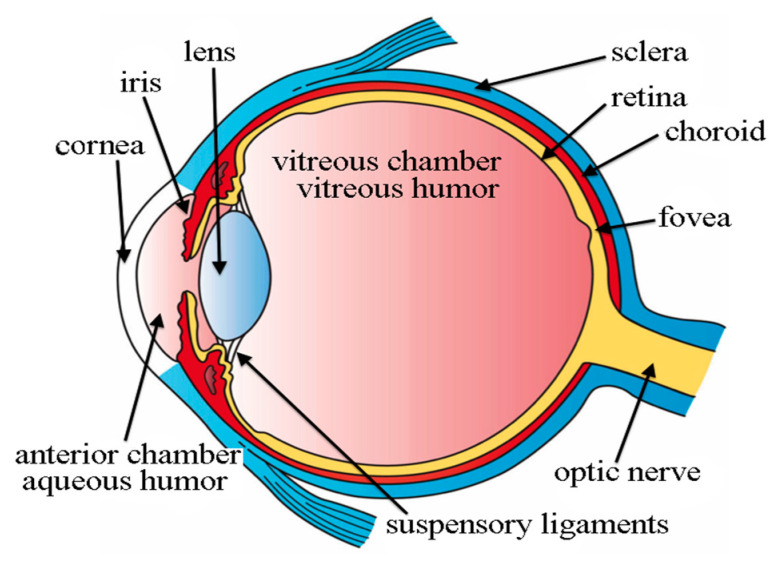
Basic Structure of Eye which are used in the diagnosis of AD [17].

Research into Alzheimer’s disease is increasingly relying on optical coherence tomography (OCT) and optical coherence tomography angiography (OCTA). AD sufferers’ eyes have thicker retinal and choroidal layers than those of cognitively normal individuals. OCTA provides voluminous data utilised in vascular density deficits in the pathophysiology of neurodegenerative diseases [15]. Alzheimer’s and moderate cognitive impairment (MCI) patients had statistically significant reductions in rNFL zone and retinal thickness mean peripapillary thickness. Basic structure of eye for understanding is shown in Figure 2. In 2017 Dutch study including 2124 people, researchers compared OCT measures of retinal sublayer size with brain MRI data. Grey and white matter volumes were considered to be decreased in the retinal sub layers rNFL, Ganglion cell zone, and inner plexiform zone rNFL; total macular and ganglion cell zone thinning were all measured using 3T brain MRI and optical coherence tomography (OCT) [16]. 

A study released in 2019 found that the size of the inner retinal zone decreased in patients with Alzheimer’s disease (AD). A major prospective multicenter study in the United Kingdom involving OCT imaging and successive volumes of four cognitive tests included more than 32,000 participants. A primitive biomarker or biomarker for dementia and other neurodegenerative diseases, the thickness of the RNFL, could be utilized as it was found to be nearly twice more likely to be found in the bottom 40% of participants than in those with thicker rNFL [18]. Significant Findings of various authors are listed below in Table 1. 

### 2.1. Image Reconstruction-Recognizing Objects over Background Luminance

Image reconstruction converts raw data into pictures that may be used to evaluate clinically based MRIs. In this process, the picture quality is affected by a number of signal processing steps. OCT images are subjected to a high level of noise since they rely on interferometry. To obtain a clean, high-resolution image, various techniques were adopted based on the OCT image. Significant improvements in noise reduction and image reconstruction were achieved using local statistical algorithms, an adaptive noise smoothing filter, and an optimal MMSE filter [31,32,33]. Second-order tensor-based total variation was utilised to suppress artefacts and protect retinal layers in the low-rank approximation model [34]. Accurate processing of visual and neurological information is required to distinguish between objects at different spatial frequencies in the contrast sensitivity test. Even when their vision is 20/20 Snellen, people with severe Alzheimer’s disease can see a “haze” or “mist” in their vision [35]. In contrast to age-matched control patients, this haziness in vision is caused by a loss of contrast sensitivity. Contrast sensitivity suffers more than Snellen resolution acuity from the degenerative processes that occur in AD [36,37]. Alzheimer’s patients were found to be less sensitive to contrast across all spatial frequencies than healthy elderly people [38]. This decrease in contrast sensitivity may be the cause of the apparent reading difficulties in AD patients, even though their acuity levels are still quite high [39]. Researchers tested the contrast sensitivity of people with Alzheimer’s disease and healthy older and younger control groups [39] by exposing them to single letters of changing spatial frequencies and contrast levels. However, when it came to spotting single letters in high contrast, the AD group scored worse than its older control group. An eye exam can reveal a biomarker of neurodegeneration that reduces contrast sensitivity in Alzheimer’s disease, according to the research quoted above.

### 2.2. Image Filtering-Biomarkers for AD Diagnosis Based on Structural Changes in the Retina, Optic Nerves, and Lenses

Filters are mostly employed in image processing to reduce the picture’s high-frequency content. Biomarkers for Alzheimer’s disease diagnosis may be found in the eye, which has piqued researchers’ interest. Hinton [40] was the first to discover that Alzheimer’s patients had ONH (optic nerve hypoplasia) and retinal abnormalities. Four Alzheimer’s patients were discovered to have a thinning of the ONH, as well as a loss in ganglion cells. The peripapillary RNFL of Alzheimer’s patients has lately thinned, as reported by studies utilising in vivo optical imaging methods. The RNFL was used to evaluate Alzheimer’s disease biomarkers in vivo. 

Rapid and minimally invasive retinal cross-sectional imaging is possible with OCT. Alzheimer’s disease diagnosis and therapy will benefit greatly from the use of an OCT. Optical coherence tomography (OCT) was utilised to test patients with amnestic mild cognitive impairment (MCI). Alzheimer’s disease (AD) patients had poorer maculas, particularly in the inferior, temporal, and nasal retinal regions, according to recent studies. Danesh-Meyer et al. [41] investigated the ONHs of 40 AD persons using fundoscopy. The AD group had a threefold greater optical cup-to-disc ratio than the control group. The unique deficits in the inferior visual field found in persons with AD may be caused in part by the loss of retinal ganglion cells. The inverted retinal area that corresponds to visual space may promote neuronal loss in the superior retina.

Biological factors are employed as biomarkers in order to identify a subclinical manifestation of the illness or a stage of the ailment. Amyloid positron emission tomography (PET), tau PET, and MRI help identify severity stages. Studies have linked retinal degeneration to Alzheimer’s disease, although the evidence is equivocal at best. Kergoat et al. [42] used scanning laser polarimetry (SLP) to assess the RNFL thickness in 30 Alzheimer’s patients and 30 healthy controls. Alzheimer’s patients and healthy controls present no considerable variations in the placement of nerve fibres. The RNFL appears to be untouched in the beginning stages of Alzheimer’s disease, according to these results. HRT and GDx nerve fibre analyzer scanning laser polarimetry results for AD patients reveal no significant differences; however, OCT data show a significant difference. The earlier the investigation, the more likely it was to make use of an older piece of gear.

### 2.3. Image Segmentation-Optic Nerve Fibre Imaging and AD

Segmenting an image into distinct areas or categories is the process of identifying separate items or sections of an object in the picture, as shown in Figure 2. By comparing the structural and functional variation of the brain, MRI imaging segmentation aids in the process of identifying the affected region. In retina, optical nerve fibre thickness was considerably lower in Alzheimer’s disease patients and controls than in healthy individuals [43]. Researchers studied 63 people aged 63–77 who had Alzheimer’s disease that was only mild to moderate in severity. Iseri and colleagues found that the macular volume of Alzheimer’s patients was significantly less than that of healthy participants [44].

**Figure 3:**
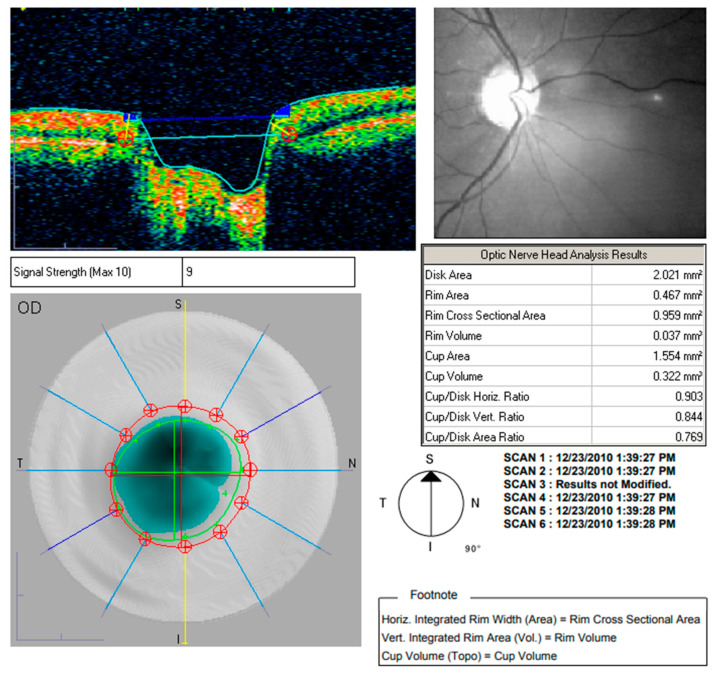
Optic nerve head analysis using Optic Disc.

**Figure 4:**
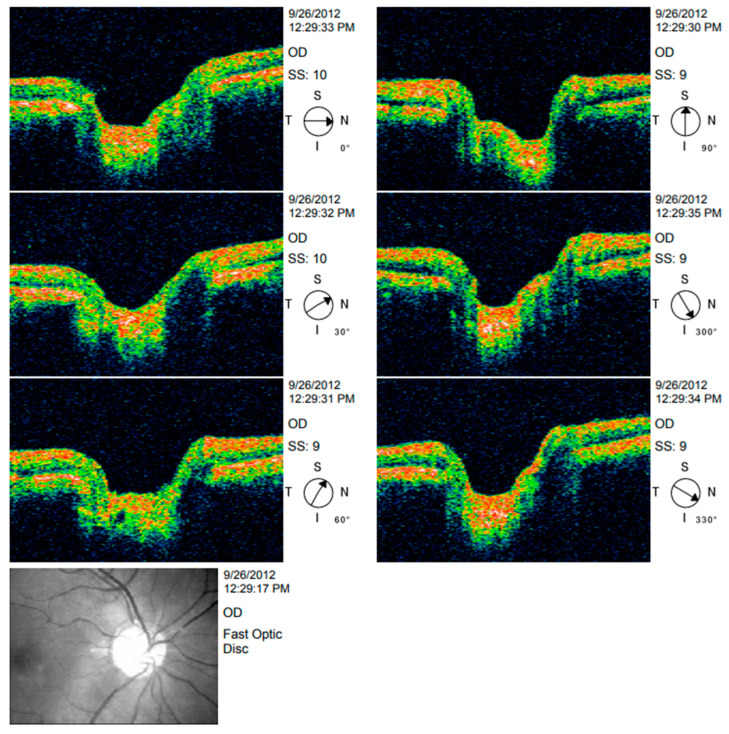
OCT image analysis.

Both macular and nerve fibre zone thinning have been observed in those with Alzheimer’s disease (AD) [44]. Valenti also found the same thing with an OCT [45]. Only the OCT group of glaucoma patients had abnormal nerve fibre zone thicknesses. The average thickness of the superior optic nerve fibre zone in glaucoma patients was 106 millimetres; in AD patients, it was 90 mm, and in healthy participants, it was 115 mm. An undiagnosed ocular condition such as glaucoma did not cause the OCT abnormalities found in this study. Instead, they were observed to be specific to Alzheimer’s disease. Alzheimer’s patients in the milder and more moderate phases of the disease might both take part in the research. The comparison of various sectors is shown in Table 2 [46]. Optic nerve head analysis of a retina using Optic Disc and the OCT image analysis for RNFL is shown in Figure 3 and Figure 4 respectively for references.

### 2.4. Image Registration

Importing disparate data sets into a single coordinate system is called image registration. Ten patients with Alzheimer’s disease had a lower response to the magnocellular route when positron emission tomography (PET) was employed, compared with participants without the condition [47]. As the temporal frequency increased, so did the frequency of the stimuli. Frequencies over 25 Hz were associated with greater differences between AD and age-matched controls than lower frequencies. A person with intermediate Alzheimer’s disease showed a diminished response in the magnocellular circuit. Persons with Alzheimer’s perform worse on FDT visual field tests, which employ a high-frequency target [48].

**Figure 5:**
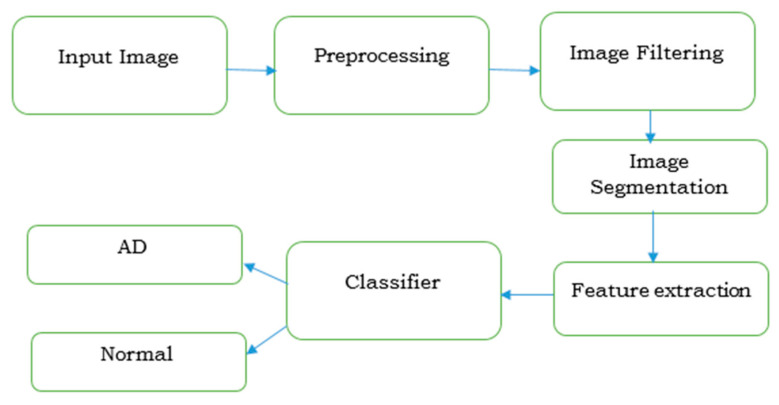
Imaging procedure for diagnosing Alzheimer’s disease.

The steps involved in the diagnosis of AD by imaging techniques is discussed in Figure 5. The severity in identification is carried out by extracting its features by machine learning or deep learning algorithms.

## 3. Discussion

According to the results of the study, preclinical Alzheimer’s disease patients may show signs of retinal vascular and architectural abnormalities. According to this study, retinal neuronal loss and vascular alterations may occur earlier than previously thought. Alzheimer’s disease-related cerebral neuronal death exhibits a similar phenomenon in that it occurs long before any symptoms appear. Because of numerous confounding factors, further research is required to determine if this finding is beneficial in the diagnosis of preclinical Alzheimer’s disease.

Biomarker-positive subjects were reported to have thinning of the inner fovea in previous research utilising OCT and primitive autopsy techniques. To be fair, although illness status differs significantly across groups statistically, there is so much overlap in distribution that these results are practically useless. This also saw a decrease in foveal vasculature in the biomarker-positive group, which led to an increase in the FAZ (foveal avascular zone). Brain hypoperfusion has been linked to vascular dysfunction in people with MCI and Alzheimer’s disease since 2007. Previous in vivo and autopsy studies have linked amyloid and collagen build up in cerebral capillaries to cell death and vessel dropout. Although there is a possibility that the retinal vasculature, like the cerebral vasculature, may be affected in the same way as the cerebral vasculature in the progression of AD, this is merely an observational study, not an investigation of the underlying causes.

Retinal degeneration caused by Aβ build up in the retina may also contribute to FAZ enlargement in those with preclinical Alzheimer’s disease. Most of the research on postmortem tissue from Alzheimer’s patients shows the presence of Aβ plaques in the interior region of postmortem tissue, although some sources have stated that these deposits are only found in superior retinal tissue. Other research in human tissues, on the other hand, has failed to detect retinal Aβ [49], while others imply that accumulation may be more substantial. No conclusive conclusion can be drawn because there are only a few relevant studies and their methods are so different.

As the name suggests, optical coherence tomography (OCT) uses light to create images. A Topcon OCT Model 3D OCT-1000 (Japan, Topcon) was utilised to quantify the size of the macula and the retinal and peripapillary nerve fibre zone (RNFL) in order to obtain an accurate reading. The thickness of the RNFL was measured three times at each location. For statistical purposes, the mean values were used. The same optometrist performed all of the examinations in the same manner and with the same equipment (ESG).

The MMSE scores of patients with moderate Alzheimer’s disease were the highest ever recorded (25.18 3.80). This group of patients was just beginning to show signs of illness. A score of 19.89 2.76 on the MMSE indicated that they were capable of following directions and correctly completing the exams. When deciding which tests to use, consideration was given to cognitive decline and a possible nominative insufficiency. Patients with dementia obtained better VA test results when the letters were isolated because they were dispersed that way [50]. The test values are displayed in Table 3. The CSV-1000E was used to measure the CS, which does not require the patients to speak the results. As a result, the outcomes were less dependent on the VA levels [36]. Because verbalization was not required for its manifestation, the Roth 28-hue test was used to examine colour perception. The patient’s participation in OCT measurements of retinal thickness was modest, and the patient was simply asked to maintain one eye open and stare fixedly at the fixation stimulus.

**Figure 6:**
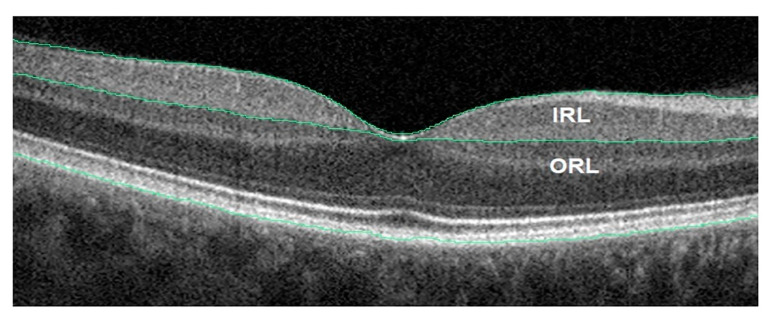
AD patient OCT image. Inner and outer retinal layer (IRL and ORL) [52].

**Figure 7:**
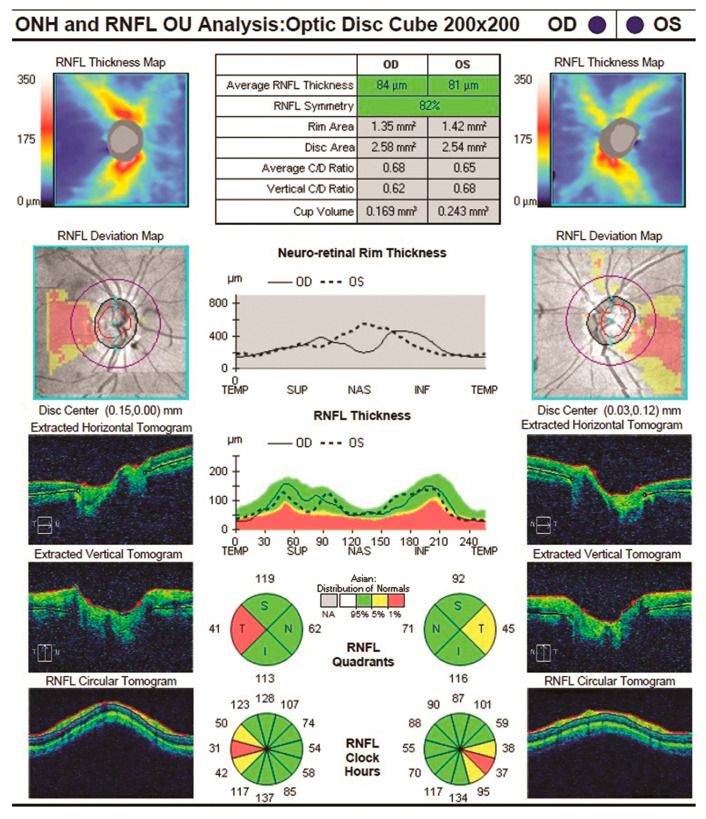
RNFL of an Alzheimer’s disease patient [23].

Using the latest OCT technology, a decrease in the RNFL + GCL complex thickness in AD patients can be observed. The retina’s outer layers were unaffected by the disease. Inner and outer retinal layer of an AD patient OCT image is shown in Figure 6. The GCL-IPL complex was also found to have significantly decreased. The thickness of the complex was found to have a positive connection with the MMSE scores in Alzheimer’s disease [51]. RNFL thickness was found to be less of a prognostic factor than the complex’s association with a decrease in macular blood flow velocity. Alzheimer’s disease individuals with moderate dementia had their RNFL, GCL, and IPL all thin. Figure 7 shows the spectral domain OCT of the RNFL in a patient with AD. The Rotterdam study discovered that the internal retinal layers were weakening. As a result of their research, they discovered that patients have a reduced volume of the brain’s hippocampus, which is one common clinical feature. OCTA helps to find the retinal vascular density deficits, which are used in the diagnosis of Alzheimer’s disease [52].

## 4. Conclusions

To diagnose Alzheimer’s, neuroimaging and CSF tests are used to interpret the symptoms and signs found in the CSF as well as brain neuroimaging. Recent advances in brain imaging have made it possible to make more precise diagnoses, but these methods are expensive and scarce, making it difficult to conduct population-wide screenings. Therefore, with new, more primitive diagnostic approaches that are more precise, there is an urgent need for diagnostic tools that are less expensive and better able to distinguish among Alzheimer’s disease and other types of neurodegenerative diseases. Eye disease can be diagnosed and treated earlier if new biomarkers for the disease are discovered and developed. Retinal amyloid plaques, which are the hallmarks of AD, have been documented by several studies recently. This suggests that a simple, non-invasive eye scan could be used to identify the disease. The optical retinal imaging platforms developed by several labs are being tested to identify amyloid plaques in Alzheimer’s patients’ retinas. Early Alzheimer’s disease detection and therapeutic drug efficacy monitoring is made easier with these cutting-edge imaging tools that target critical cellular pathways. To detect Alzheimer’s disease (AD) using biomarkers, which have not yet been proven in clinical settings, is a major challenge. Many factors contribute to this, such as the researchers’ lack of expertise in interpreting the enormous amounts of data generated by these eye scans, a lack of specificity in these biomarkers, and a lack of ethnic variation in the data. People of different nationalities and origins need more validation studies to be conducted. If amyloid plaques in the retina have already been detected by the time the disease has progressed, ocular biomarkers may be used too late. Research into how retinal cells die in ocular neurodegenerative disorders is leading scientists to a new generalisation about these illness. Even though the exact cause of AMD, glaucoma, and Alzheimer’s disease is still a mystery, researchers can learn more about the pathogenesis of these diseases by comparing their symptoms. Moreover, it could lead to a better understanding of AD diseases and treatments, as well as the brain and cellular pathways in other retinal degenerative diseases in the future.

## Figures and Tables

**Table 1:** Significant Findings.

Year, Authors [Ref]	Significant Finding
Yan et al., 2021 [19]	rNFL and retinal vascular density were considerably lower in persons with Alzheimer’s disease than in healthy controls. Some patients’ cognitive dysfunction has been linked to a decrease in retinal vessel density.
Mei et al., 2020 [20]	Investigation of the link between the retinal and cerebral Aβ content in APP mice. In the retina, curcumin can stain Aβ, but it has been found to suppress the level of this staining compound.
Chua et al., 2020 [21]	AD patients’ superficial and deep capillary plexus showed considerably decreased vascular density compared with healthy controls.
Wu et al., 2020 [22]	OCT-A on AD patients revealed significantly lower deep retinal capillary plexus micro vascular densities than on matched controls.
Kim et al., 2019 [23]	Both individuals with serious Alzheimer’s disease and those with mild to medium Alzheimer’s disease had thinning of the rNFL assessed by OCT.
Zabel et al., 2019 [24]	Comparing patients with AD to healthy controls, their rNFL was significantly thinner, but this was not statistically significant. SD-OCT observed changes that were not specific.
Ko et al., 2018 [18]	Researchers found that patients without the neurodegenerative disease had lower cognitive function and the possibility of cognitive reduction as a result of the presence of thinning rNFL.
Sanchez’ et al., 2018 [25]	For both cognitively healthy and Alzheimer’s disease persons, the thickness of the peripapillary rNFL is the same.
Koronyo et al., 2017 [26]	Curcumin fluorochrome was used to detect a deposit at the retinal level. Imagery of the retina A was captured using solid lipid curcumin and an adapted scanning laser eye scope.
Den Haan et al., 2017 [27]	Mild cognitive impairment (MCI) and Alzheimer’s disease (AD) patients exhibit reduced retinal nerve fibre zone and macular thickness compared with healthy controls, respectively.
Mutlu et al., 2017 [28]	Gray and white matter volumes were found to be less when the RNFL and GCL were smaller as measured by OCT.
Casaletto et al., 2017 [16]	On OCT, overall macular and macular ganglion cell volumes were shown to decrease.
Jiang et al., 2016 [29]	A meta-analysis of five studies with few sample sizes was unable to make any conclusions about pathological retinal degeneration.
Pillai et al., 2016 [30]	There is no variation in rNFL, GCL, or macular volume on OCT in Alzheimer’s disease patients compared with healthy control subjects.
Koronyo Hamaoui et al., 2011 [4]	It was discovered that systemic administration of curcumin caused the development of retinal postmortem eyes of Alzheimer’s patients to show a buildup of plaques. These plaques can be seen and accumulated at a primitive phase of illness.

**Table 2:** The thickness of the retinal nerve fibre layer in various parts of the optic disc for 10 patients is listed out.

Patient	Mean	NS	N	NI	TI	T	TS
1	90.0 ± 8.5	75.0 ± 1.4	46.0 ± 9.9	111.5 ± 21.9	143.5 ± 9.2	73.0 ± 1.4	153.0 ± 17.0
2	102.0 ± 8.5	105.5 ± 3.5	60.5 ± 6.4	112.0 ± 7.1	166.5 ± 13.4	77.5 ± 17.7	157.5 ± 0.7
3	100.0 ± 0.0	99.0 ± 4.2	99.5 ± 0.7	127.5 ± 2.1	130.5 ± 2.1	61.5 ± 0.7	122.5 ± 0.7
4	99.0	124.0	92.0	127.0	134.0	58.0	108.0
5	110.0 ± 5.7	116.5 ± 4.9	81.0 ± 5.7	129.0 ± 4.2	148.5 ± 2.1	86.5 ± 13.4	151.0 ± 5.7
6	95.5 ± 4.9	110.5 ± 16.3	66.0 ± 2.8	91.5 ± 21.9	143.5 ± 23.3	68.0 ± 1.4	148.5 ± 14.8
7	108.0 ± 1.4	111.5 ± 16.3	87.0 ± 0.0	125.5 ± 4.9	147.5 ± 3.5	77.5 ± 4.9	139.0 ± 0.0
8	85.0 ± 1.4	85.5 ± 4.9	55.0 ± 1.4	73.0 ± 5.7	129.0 ± 9.9	81.5 ± 20.5	118.5 ± 7.8
9	90.5 ± 2.1	91.5 ± 7.8	67.0 ± 2.8	80.5 ± 2.1	147.0 ± 4.2	66.0 ± 0.0	139.0 ± 11.3
10	93.0 ± 0.0	110.0 ± 0.0	66.5 ± 0.7	124..0 ± 5.7	141.5 ± 7.8	59.5 ± 0.7	114.5 ± 0.7

Mean and standard deviation intervals for a few patients are shown here. Labelled sectors are defined to be N—nasal, NS—nasal Superior, NI—nasal inferior, TI—temporal inferior, T—temporal, and TS—temporal superior.

**Table 3:** Median data and *p*–value of the psychophysical tests [51].

		Control	Mild AD	Moderate AD	Mild AD vs. Control		Moderate AD vs. Control		Mild AD vs. Moderate AD	
		(*n* = 40)	(*n* = 39)	(*n* = 18)	% Difference	*p*-Value	% Difference	*p*-Value	% Difference	*p*-Value
Visual Acuity (dec)		1.00 ± 0.10	0.90 ± 0.20	0.90 ± 0.30	−10.00	<0.001 **	−10.00	0.003 **	0.00	0.921
Constrast Sensitivity (cpd)	3	1.63 ± 0.29	1.49 ± 0.46	1.49 ± 0.29	−8.59	<0.001 **	−8.59	0.009 **	0.00	0.422
	6	1.84 ± 0.44	1.70 ± 0.33	1.55 ± 0.28	−7.61	<0.001 **	−15.76	<0.001 **	−8.82	0.373
	12	1.54 ± 0.29	1.25 ± 0.32	1.16 ± 0.30	−18.83	<0.001 **	−24.68	<0.001 **	−7.20	0.599
	18	1.10 ± 0.29	0.64 ± 0.56	0.64 ± 0.57	−41.82	<0.001 **	−41.82	<0.001 **	0.00	0.781
Number of Errors										
	Total	5.0 ± 5.0	7.0 ± 4.0	12.00 ± 8.0	40.00	0.034 *	140.00	<0.001 **	71.43	0.001 **
	Tritan	2.0 ± 2.0	3.0 ± 3.0	6.0 ± 5.0	50.00	0.002 **	200.00	<0.001 **	100.00	0.017 *
	Deutan	1.0 ± 3.0	3.0 ± 3.0	5.0 ± 3.0	200.00	0.003 **	400.00	<0.001 **	66.67	0.002 **
PDT		14.00 ± 2.00	12.00 ± 4.00	11.50 ± 3.0	−14.29	<0.001 **	−17.86	<0.001 **	−4.17	0.650

Median ± interquartile range, * *p* < 0.05, ** *p* < 0.01, Mann–Whitney U test. AD, Alzheimer’s disease; vs.: versus; dec: decimal scale; cpd: cycles per degree; PDT: perception digital test.

## Data Availability

Not applicable.
